# Management of Sprengel deformity by modified Woodward approach and outcome: a case series and literature review

**DOI:** 10.1097/MS9.0000000000002763

**Published:** 2025-01-09

**Authors:** Abhishek Pandey, Sabik R. Kayastha, Prasamsa Pande, Prabhat Silwal, Prashant Upadhyaya, Archana Pandey, Suraj Keshari, Sanjay Dhungana, Yadvinder Singh

**Affiliations:** a Dhulikhel Hospital, Kavre, Bagmati, Nepal; b Department of Orthopedics & Traumatology, Kathmandu University School of Medical Sciences, Kavre, Bagmati, Nepal; c Kathmandu Medical College, Kathmandu, Bagmati, Nepal; d Nepal Medical College and Teaching Hospital, Kathmandu, Bagmati, Nepal

**Keywords:** case series, Klippel-Feil syndrome, modified Woodward approach, omovertebral bone, Sprengel deformity

## Abstract

**Introduction:**

Sprengel deformity (SD) is a rare congenital deformity consisting of elevation of the scapula, leading to limited range of motion and a visible lump in severe cases. Klippel-Feil syndrome is a rare congenital abnormality characterized by the triad of cervical synostosis, low posterior hairline, and short neck. It is often associated with SD.

**Case presentation:**

We present two cases of SD of a 7-year-old boy and a 10-year-old boy. They were both born with the abnormality and complained of limited range of motion in the affected shoulder.

**Clinical discussion:**

Patients with less severe forms of SD can lead a normal life with supportive treatment, and it is not connected with cognitive impairment; however, there may be a limited range of motion and cosmetic concerns. In severe cases, treatment entails operative therapy with corrective placement of the scapula and resection of the omovertebral band.

**Conclusion:**

Patients with SD require supportive physiotherapy from a young age. Severe cases need operative treatment at a young age. The modified Woodward approach may be an excellent surgical option to correct the deformity.

## Introduction

Sprengel deformity (SD), or congenital high scapula, is a rare congenital skeletal disorder with elevation of the scapula due to failure to descend during fetal development^[1]^. It is the most common congenital anomaly of the scapula^[2]^. There is often restriction of scapular movement in SD due to abnormal connection with the spine, dysplasia, and/or disorientation of the shoulder blade^[1,3-5]^.

Klippel-Feil syndrome (KFS) is a congenital anomaly associated with cervical synostosis; it characteristically comprises the triad of cervical vertebral body fusion, low posterior hairline, and short neck with limited range of motion. KFS is seen in approximately 1 in 40 000–42 000 births and is associated with SD between 7% and 42% of cases^[6,7]^.

SD can occur as an isolated defect or in conjunction with other abnormalities^[4]^. One of the abnormalities associated with SD is a bony, cartilaginous, or fibrous connection from the superomedial border of the scapula to the cervical spine, an omovertebral fusion^[8]^. This connection has been reported in approximately one-third of patients with SD^[9,10]^.

Surgical correction is the preferred treatment of choice in SD for two reasons: functional and cosmetic^[3]^. The various options in deformity correction include resection of bony prominence,^[10]^ lowering of the scapula by detachment of muscles at their scapular insertion,^[11]^ lowering of the scapula by detachment of muscles at their spinal origin,^[12]^ lowering of the scapula by performing scapular osteotomy,^[13]^ or a combination of options.

This prospective single-center case series is being reported due to its rarity and to highlight the successful outcome achieved using the modified Woodward approach. Furthermore, there is also a brief review of KFS and SD with the different diagnostic and corrective modalities that can be applied for diagnosis and treatment^[14]^.

## Case 1

### Patient information

This 5-year-old boy presented to the outpatient department at our tertiary care center with a history of shoulder asymmetry and a short neck since birth. His medical and surgical history was uneventful, and there was no history of trauma in the patient. There was no significant drug or allergy history. No similar conditions had been reported in the family.

### Clinical findings

**Figure 1.**
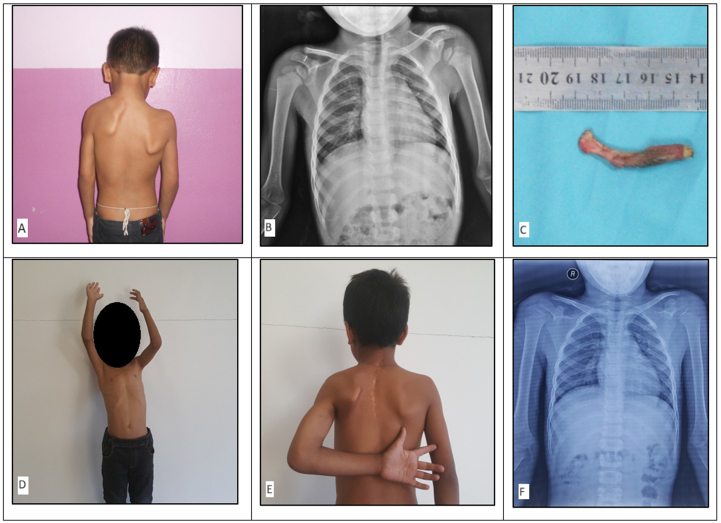
(A) Preoperative picture showing the difference in the heights (left above right) of the shoulder as seen on gross inspection. (B) Preoperative plain radiograph of anteroposterior view of the chest showing the difference in the position of the scapula (left above right). (C) Postoperative omovertebral band specimen after excision as compared to a standard metric scale (~5 cm). (D) Postoperative range of arm flexion. (E) Postoperative range of internal rotation of arm. (F) Postoperative plain radiograph showing the correction of scapular elevation with a decreased height of the left scapula.

Clinically, restricted neck mobility and a high left scapula were observed. The left shoulder joint was elevated by 3 cm in comparison to the contralateral side (Fig. 1A), and an omovertebral bone was palpable. Examination of the patient’s back demonstrated a higher left scapula with a hard prominence palpable at the cervicothoracic junction. The range of motion of the neck and left shoulder was restricted. Neurological examination showed intact cranial nerves and no motor deficit. Blood tests and urinalysis were normal. An ultrasonogram of the abdomen and pelvis and an echocardiogram were also performed and ruled out other congenital anomalies.

### Diagnostic assessment and interpretation

**Figure 2.**
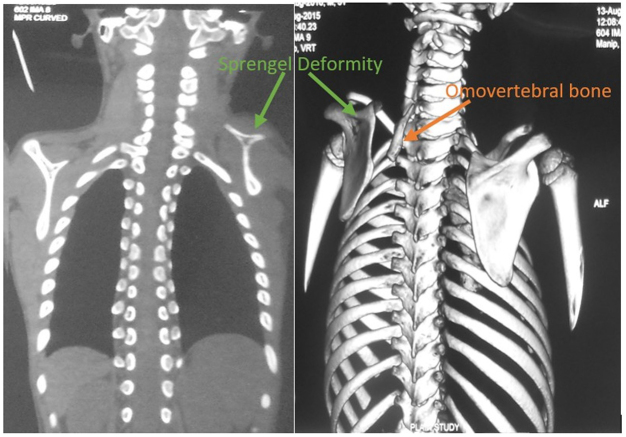
These findings on CT images (coronal and Volume Rendering Technique) are consistent with a left-side elevated scapula (green arrow) with an omovertebral bone (orange arrow) (a bar connecting the scapula with the cervical spine) at the level of the C6 vertebra.

Initial plain radiographs of anteroposterior (Fig. 1B) and lateral views of the chest demonstrated elevation of the left scapula. Subsequent CT imaging revealed an omovertebral bone at the level of the C6 vertebrae. These findings were interpreted as omovertebral bone in combination with unilateral SD (Fig. 2). Resection of the omovertebral bone was indicated.

### Intervention

The patient was advised to undergo physiotherapy with a concurrent prescription of pain medication. No improvements in the patient’s condition were observed. The patient was planned for operative therapy due to no improvement with non-surgical management paired with the presence of grade 3 SD according to the Cavendish classification, both being indications for surgical management. The surgery was performed by two senior orthopedic surgeons with assistance from an orthopedic medical officer, anesthesiologists, and nurses.

The patient was placed in a supine position. After painting and draping, an incision of about 5 cm was made over the left clavicle. Subcutaneous tissue and subperiosteal clavicular resection were done, and the periosteum was repaired. The coracoid process was identified, and the anterior portion of the coracoid process was resected, followed by closure of the wound. Then the patient was repositioned in a prone position, and a midline incision of about 12 cm was made from lower cervical level to middorsal level. Levator scapulae and trapezius muscles were detached from the spinous processes. The omovertebral bar was attached over the infraspinous region and was resected measuring ~5 cm × 0.5 cm (Fig. 1C). Rhomboid minor and major muscles were detached from the spinous process of the scapula. The scapula was mobilized caudally along with abduction and external rotation of the left upper limb. Then, the scapula was secured in a pocket of trapezius by suturing with ribs. The trapezius was repaired. The drain was kept and the wound was closed. Postoperatively, the left shoulder joint was elevated by less than 1 cm (Fig. 1E) in comparison to the contralateral side, and an omovertebral bone was palpable. Philadelphia brace was applied.

### Follow-up and outcomes

The wound dressing was changed on alternate days, the drain was removed on the fourth postoperative day, and the patient was discharged on the sixth postoperative day with a healthy wound. The Philadelphia brace was continued for a month. The patient was prescribed an oral antibiotic (oral Cefuroxime 500 mg twice daily for 7 days) and an oral analgesic medication on discharge. The sutures were removed on the 10th postoperative day, and the patient has been followed up every 3 months. The patient reported adhering to the prescribed medication and continuing on-and-off over-the-counter analgesic use until 3 months following surgery. There were no intraoperative or postoperative complications. On the 14th day follow-up, plain anteroposterior and lateral chest radiographs were taken (Fig. 1F), and physiotherapy commenced, starting with wound physiotherapy for the first 2 weeks and gradually shifting toward range of motion physiotherapy following the third week as advised by the physiotherapist. The patient continued physiotherapy and came in for follow-up visits every 3 months until the 1-year postoperative milestone was reached.

We have continued follow-up visits with the patient every 6 months. Each follow-up includes a thorough clinical examination of the wound site and functionality of the affected shoulder. The patient has completed 3 years of postoperative follow-up visits and has been advised to come for follow-up if any concerns arise.

## Case 2

### Patient information

This 8-year-old boy presented to the outpatient department at our tertiary care center with a history of shoulder asymmetry and decreased range of motion of the left shoulder associated with pain over the left scapular region since birth. Otherwise, his medical and surgical history was uneventful, and there was no history of trauma in the patient. There was no significant drug or allergy history. No similar conditions had been reported in the family.

### Clinical findings

**Figure 3.**
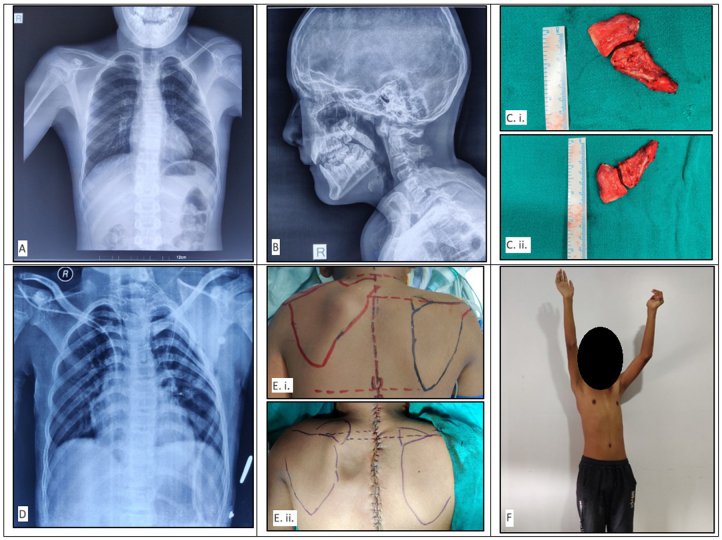
(A) Preoperative plain radiograph showing the difference in height of the scapula (left higher than right). (B) Preoperative picture showing fusion of C2–C3 vertebral body, C3–C4 vertebral spine, and C5–C6 vertebral spine. (C): (i) Postoperative omovertebral band specimen after excision as compared to standard metric scale (length ~ 6 cm); (ii) postoperative omovertebral band specimen after excision as compared to standard metric scale (breadth ~ 3 cm). (D) Postoperative plain radiograph showing the correction of scapular elevation with a decreased height of the left scapula. (E): (i) Preoperative gross inspection of the back of the patient with bilateral scapula marked showing difference in height; (ii) postoperative gross inspection of the back of the patient with bilateral scapula marked showing improvement in the height discrepancy of the left scapula with dimpling at the site of rhomboid reconstruction. (F) Postoperative range of arm flexion.

Clinically, low posterior hairline, restricted neck mobility, and high left scapula were observed. The left shoulder joint was elevated by 5 cm (Fig. 3E i) in comparison to the contralateral side, and an omovertebral bone was palpable. Palpation of the patient’s back demonstrated a higher left scapula with a hard prominence palpable at the cervicothoracic junction, while the spine was normal. The range of motion of the neck and left shoulder was restricted. Neurological examination showed intact cranial nerves and no motor deficit. However, the patient complained of pain, likely cervical myelopathy, on abduction of the left arm. Blood tests and urinalysis were normal. An ultrasonogram of the abdomen and pelvis and an echocardiogram were also performed and ruled out other congenital anomalies.

### Diagnostic assessment and interpretation

Initial plain anteroposterior and lateral radiographs demonstrated elevation of the left scapula (Fig. 3A). Furthermore, vertebral body fusion of C2-3 and C4-6 was observed (Fig. 3B). Subsequent CT imaging revealed an omovertebral bone at the level of the C6 vertebrae. These findings were interpreted as omovertebral bone in association with unilateral SD and KFS. The patient was diagnosed as grade 3 SD according to the Cavendish classification. Resection of the omovertebral bone was indicated. Deformity correction by the modified Woodward procedure was planned.

### Intervention

The patient was advised for physiotherapy with a concurrent prescription of pain medication. No improvements in the patient’s condition were observed. The patient was planned for operative therapy due to no improvement with non-surgical management paired with the presence of grade 3 SD, both being indications for surgical management. The surgery was performed by two senior orthopedic surgeons with assistance from an orthopedic resident, an orthopedic medical officer, anesthesiologists, and nurses.

The patient was placed in a supine position. After painting and draping, an incision of about 5 cm was made over the left clavicle. Subcutaneous tissue and subperiosteal clavicular resection were done, and the periosteum was repaired. The coracoid process was identified, and the anterior portion of the coracoid process was resected, followed by closure of the wound. Then the patient was repositioned in a prone position, and a midline incision was made from lower cervical level to middorsal level. Levator scapulae and trapezius muscles were detached from the spinous processes. An omovertebral bar with a fibrous band was observed, attached over the infraspinous region, and was resected following isolation. It was measured to be approximately 6 cm × 3 cm (Fig. 3C i and ii). Rhomboid minor and major muscles were detached from the spinous process of scapula. Scapula was mobilized caudally along with abduction and external rotation of the left upper limb. Then, the scapula was secured in a pocket of trapezius by suturing with ribs. Trapezius was repaired. Drain was kept, and the wound was closed. Postoperatively, the left shoulder joint was elevated by 1 cm (Fig. 3E ii) in comparison to the contralateral side, and an omovertebral bone was palpable. Philadelphia brace was applied.

### Follow-up and outcomes

The wound dressing was changed on alternate days, the drain was removed on the fifth postoperative day, and the patient was discharged on the sixth postoperative day with a healthy wound. Philadelphia brace was continued until 30 days following surgery. The patient was prescribed an oral antibiotic (oral Cefuroxime 500 mg twice daily for 7 days) and an oral analgesic medication on discharge. The sutures were removed on the 12th postoperative day, and the patient has been followed up at 2 weeks and every subsequent 3 months until 1 year from the operation. On the 14th day follow-up, plain anteroposterior and lateral chest radiographs were taken (Fig. 1F), and physiotherapy commenced starting with wound physiotherapy for the first 2 weeks and gradually shifting toward range of motion physiotherapy following the third week as advised by the physiotherapist. The patient reported adhering to the prescribed medication and continuing on-and-off over-the-counter analgesic use until 2 months following surgery. There were no intraoperative or postoperative complications. The patient has continued physiotherapy and come in for follow-up visits every 3 months until the 1-year postoperative milestone was reached.

We have continued follow-up visits with the patient every 6 months. Each follow-up includes a thorough clinical examination of the wound site and functionality of the affected shoulder. At the time of the last visit, the wound site showed signs of a hypertrophic scar for which the patient was referred to dermatology. The patient has completed 1 year of postoperative follow-up visits and has been advised to continue a 6-month follow-up regimen until 3 years following surgery.

## Discussion

This case series involves two patients who presented with complaints of shoulder asymmetry and decreased range of motion in the affected shoulder since birth. The patients were 5 and 8 years old at the time of presentation, respectively. Clinical assessment and CT images confirmed SD in both patients. Operative correction of the deformity was planned using the modified Woodward approach and performed in both patients. Both patients have no intraoperative or postoperative complications and have reduced scapular misalignment and greater range of motion of the shoulder at 3-year and 1-year follow-up visits, respectively. This study suggests that the modified Woodward approach may be regarded as a successful operation for SD and operative correction at a young age may lead to better outcomes.

In 1863, Eulenberg was recognized as having written the first account of congenital elevation of the scapula. Since Sprengel documented four examples of the illness in 1891, the condition has been linked to his name^[15,16]^. The deformity was later described in several brief case studies and retrospective assessments that clarified any potential-related problems.

Normally, the scapula moves in a scapulothoracic plane during abduction. As abduction progresses, the glenoid face moves medially, then tilts upward, and finally moves up as the arm moves to maximum elevation. From 0 to 30 degrees, the scapula rotates about its lower midportion; from 60 degrees onward, the center of rotation shifts toward the glenoid; and as the scapula rotates about that area, the inferior tip of the scapula is significantly laterally displaced^[17]^.

Commonly in SD, the scapula is rotated, leading to medial displacement of the inferior tip. For this reason, the glenoid cavity faces downward, and the upwardly rotated superomedial angle appears characteristically prominent in the suprascapular region on inspection^[1]^.

Cavendish classification is used to assess the severity of deformity in SD, and it has four grades:^[18]^

1. Grade 1: Very mild disease with level shoulder joint(s) and deformity rarely visible while the patient is dressed;
2. Grade 2: Mild disease with level shoulder joint(s) and visible lump-like deformity at the web of the neck while the patient is dressed;
3. Grade 3: Moderate disease with elevated shoulder joint(s) (2–5 cm) and easily visible deformity;
4. Grade 4: Severe disease with elevation of the shoulder joint placing the superior angle of the scapula adjacent to the occiput.

Many authors refer to the Cavendish classification for surgical indication of SD, where surgery is best indicated for patients in grade 2 and grade 3^[19]^. Both of our patients fall under grade 3 of this classification, i.e., the shoulder joint is elevated by 2–5 cm compared to the opposite shoulder.

While there are no well-established indicators for surgical intervention, ROM and cosmetic appearance in relation to the Cavendish classification are commonly used to determine the necessity of surgery^[20,21]^. Surgery is indicated only in extreme situations in Cavendish grade 3 or 4^[20,21]^. A number of surgical procedures have been created with the intention of enhancing shoulder functionality and appearance^[19]^. In general, these treatments entail translating the scapula inferiorly to a more caudad position and removing the projecting section of the scapula, along with any omovertebral bones^[22]^. The Woodward and Green procedures are the most commonly performed surgical techniques^[23,24]^. Intraoperative neuromonitoring while undergoing the modified Woodward procedure for SD has been shown to be a feasible and precise means for the detection of intraoperative neurologic changes and may help in preventing brachial plexus injury^[25]^; however, we were not able to perform the surgery with concurrent neuromonitoring as this resource was not available at our institution.

The Woodward procedure involves the resection of fibroid band and/or omovertebral bone along with downward migration of rhomboids and trapezius muscles^[12]^. The modification of the Woodward procedure was proposed by Borges and Colleagues in 1996 after 31 years of the original Woodward procedure. It involves the release of muscles from their origin, relocation, and suturing distally with resection of the superomedial border of scapula. Adding morselization to the middle segment of the clavicle aims to prevent brachial plexus palsy and enhance correction^[17]^. Consequently, we performed subperiosteal resection of the middle segment of the clavicle before the actual scapular correction.

Due to the association of widened surgical scarring associated with Green’s procedure, the modified Woodward procedure remains as the reference standard^[23,26]^. Therefore, we opted to proceed with the modified Woodward procedure in our patients.

## Strengths and limitations

Favorable outcomes observed in both cases over the follow-up periods of 24 months in the first case and 12 months in the second case indicate the effectiveness of the modified Woodward approach in SD. However, the limited number of cases in this case series diminishes the generalizability of our findings. A larger dataset and comparative analysis would lead to a better understanding of this condition and its management.

## Conclusion

Here, we have delineated two cases of a rare but most common cause of congenital scapular anomaly, SD. We emphasized favorable outcomes with low complications using the modified Woodward procedure in the management of SD. Thus, the modified Woodward procedure is a viable alternative with an excellent surgical outcome for the treatment of SD.

## Data Availability

Data sharing is not applicable to this article.
